# High Expression of RECQL Protein in ER-Positive Breast Tumours Is Associated With a Better Survival

**DOI:** 10.3389/fonc.2022.877617

**Published:** 2022-05-31

**Authors:** Ardalan Mahmoodi, Ahmed Shoqafi, Ping Sun, Vasily Giannakeas, Cezary Cybulski, Sharon Nofech-Mozes, Jean-Yves Masson, Sudha Sharma, Amir Abbas Samani, Srinivasan Madhusudan, Steven A. Narod, Mohammad R. Akbari

**Affiliations:** ^1^ Women’s College Research Institute, Women’s College Hospital, University of Toronto, Toronto, ON, Canada; ^2^ Institute of Medical Science, Faculty of Medicine, University of Toronto, Toronto, ON, Canada; ^3^ Nottingham Biodiscovery Institute, School of Medicine, University of Nottingham, Nottingham, United Kingdom; ^4^ Department of Laboratory Medicine and Pathology, Faculty of Medicine, University of Toronto, Toronto, ON, Canada; ^5^ International Hereditary Cancer Center, Department of Genetics and Pathology, Pomeranian Medical University in Szczecin, Szczecin, Poland; ^6^ Sunnybrook Health Science Centre, University of Toronto, Toronto, ON, Canada; ^7^ Genome Stability Laboratory, Centre Hospitalier Universitaire (CHU) de Québec Research Center, Oncology Axis, Department of Molecular Biology, Medical Biochemistry and Pathology, Laval University Cancer Research Center, Québec, QC, Canada; ^8^ Department of Biochemistry and Molecular Biology, College of Medicine, Howard University, Washington, DC, United States; ^9^ National Human Genome Center, College of Medicine, Howard University, Washington, DC, United States; ^10^ Humber River Hospital, University of Toronto, Toronto, ON, Canada; ^11^ Dalla Lana School of Public Health, University of Toronto, Toronto, ON, Canada

**Keywords:** breast cancer, RECQL, survival, ER-positive, expression

## Abstract

**Background:**

RECQL (also known as RECQ1 and RECQL1) is a gene of recent interest in breast cancer and an association between high levels of RECQL protein in breast cancer tumour cells and good survival of patients has been reported.

**Methods:**

To validate this association, we measured the RECQL protein levels in tumours of 933 breast cancer patients using immunohistochemistry analysis and followed the patients for death from breast cancer.

**Results:**

Women with a level of RECQL protein above the 75th percentile had better 15-year disease-specific survival among ER-positive patients (62.5% vs. 48.7%, HR= 0.72, 95%CI= 0.52-0.98, p-value = 0.04), but not among ER- patients (48.9% vs. 48.0%, HR= 1.07, 95%CI= 0.67-1.69, p-value= 0.79). Among the ER-negative patients, high RECQL protein levels were associated with better survival among women who received tamoxifen treatment (67.0% vs. 51.5%, HR= 0.64, 95%CI= 0.41-0.99, p-value= 0.04).

**Conclusion:**

RECQL might be a new predictive marker for tamoxifen treatment among ER-positive patients.

## Introduction

Altered expression levels of several genes in breast cancer predict patient prognosis. A correlation has been observed between poor breast cancer prognosis and lower levels of RECQL (also known as RECQ1 and RECQL1) mRNA or RECQL protein expression (1, 2). RECQL is the smallest and most abundant member of the RecQ family of DNA helicases (3, 4). It has two main domains; the core helicase domain, involved in ATP binding and hydrolysis, and the RecQ C-terminal domain (RQC) which play an essential role in the unwinding of DNA (4–6). RECQL performs its helicase activity in an ATP-dependent manner in a 3’ to 5’ direction (7). As a helicase, it has many essential functions in DNA replication, such as maintaining the DNA replication fork progression and restarting stalled replication forks (8–11). It is also involved in maintaining genome stability, double-strand break repair, and telomere maintenance (4, 6, 12–14). In addition to its roles as a DNA helicase, RECQL is involved in branch migration of Holiday junctions and strand annealing (7). The depletion of this helicase could impair normal cellular function and lead to increased DNA damage and compromised genome stability.

An association between mutations in RECQL and breast cancer susceptibility was first reported by Cybulski et al. (15). Other studies have supported this association (16–21) but there have been negative studies as well (22–26). It has also been suggested that RECQL is a susceptibility gene for familial colorectal cancer (27).

In addition to the cancer susceptibility role, RECQL was suggested to be a prognostic marker. In a study by Arora et al. low RECQL mRNA and protein levels in breast tumours were associated with poor breast cancer prognosis (1). This correlation was observed in a second study which showed low RECQL protein levels were correlated with poor survival (HR: 2.12, p-value: 0.015) (2). Furthermore, in a recent study, the cellular mechanism by which RECQL affects ER-positive cells was proposed, wherein RECQL in cooperation with FOXA1, directly regulates the expression of the ESR1 gene which encodes the ERα protein (28).

To investigate this association further, we measured the RECQL protein levels in tumours from 933 breast cancer patients by immunohistochemistry (IHC) and analyzed their 15-year survival.

## Methods

### Study Population

This study was conducted by analyzing patients from the Banting study (29). Total of 1,601 breast cancer patients were diagnosed and enrolled between 1987 to 1999 in the Banting study and their therapy reflected the commonly used treatments during that time period. Data files and tumour samples from 933 breast cancer patients were available and analyzed in this study. Patients were followed from the date of diagnosis to the date of death from breast cancer or the date of last follow up (if alive). The average follow-up was 12.1 years (range: 0 to 31.3 years). The age at diagnosis ranged from 24 to 93 (mean 55.4 years). 55.1% of subjects were post-menopausal at diagnosis and 77.0% were ER-positive. The majority of ER-positive patients received tamoxifen (60.6%). No other endocrine therapy was used.

### Tissue Microarray and Immunohistochemistry

Tissue microarrays (TMA) were made from 0.6-mm cores sampled from the formalin-fixed paraffin-embedded tumour blocks. Each TMA contains three cores from each tumour block. Immunohistochemical staining for the RECQL protein was conducted based on a previously described method (30–33) by using a combination of Thermo Scientific Shandon Sequenza chamber system (REF: 72110017), Novolink Max Polymer Detection System (RE7280-K: 1,250 tests), and the Leica Bond Primary Antibody Diluent (AR9352), according to the manufacturer’s instructions (Leica Microsystems). The slides were dewaxed and dehydrated by Leica Autostainer XL machine. TMA sections were pretreated with sodium citrate buffer (pH 6.0) and heated for 20 minutes at 95 °C in a microwave (Whirlpool JT359 Jet Chef 1000W) for antigen retrieval. Each set of slides was incubated with primary anti-RECQL antibody (Bethyl Laboratories, catalog no. A300-450A) at a dilution of 1:1,000 for 60 minutes. For each run, positive and negative controls were included. Negative control was utilized to reassure all the staining was because of specific antibody-antigen interaction. The negative control slide had only breast tissue without adding any antibody to it. The positive control slide was a liver tissue with known expression of RECQL to control for the reactivity of the RECQL antibody and also breast cancer tissue stained with an antibody for β-globulin to control for the reactivity of immunohistochemistry enzyme.

### IHC Evaluation

After scoring whole-field inspection of the cores, intensities of the nuclear stainings were classified (0 = no staining, 1 = weak staining, 2 = moderate staining, and 3 = strong staining; **Supplementary Figure 1**). For each intensity classification, the percentage of the stained nuclei was estimated. An H-index (range 0-300) was calculated by multiplying the staining intensity score by the percentage of stained nuclei for each core. For each tumour sample, the median H-index of the three core replicates was used for the data analysis. The distribution of the median H-index for the entire cohort was shown in **Supplementary Table 1**. The mean and median of the coefficient of variation (CV) of H-index for the three cores across all samples were 10.4% and 6.9%, respectively.

### Statistical Analysis

Subjects were divided into two groups based on their RECQL protein levels (**Table 1**). We considered a high RECQL level to be one in the highest quartile (n = 205) and a medium/low RECQL level to be one in the bottom three quartiles (n = 728). This classification had the best performance in terms of distinguishing patients with poor from good survival compared to using the first quartile or median as the cut-off point. Student t tests and Fisher exact tests were used as appropriate. Estimation of cumulative survival probabilities was conducted by the Kaplan–Meier method. A log-rank test was performed for analyzing the difference between survival. A Cox proportional hazards model was used to conduct a multivariate survival analysis. Hazard ratios and 95% CIs (95% confidence intervals) for each variable were estimated. All statistical tests were two-sided. A p-value < 0.05 was considered to be significant. SAS (version 9.4) was used for the data analysis

**Table 1:** Comparison of clinicopathological characteristics between patients with medium/low versus high RECQL protein levels.

Variables	Medium/Low RECQL Protein Level N = 728	High RECQL protein Level N = 205	p-value
Age at diagnosis (years)	55.6 (24.5-93.4)	54.6 (27.8-90.1)	0.35
Follow-up (years)	12.2 (0-31.3)	11.6 (0.7-30.2)	0.31
Tumor size (mm)	27.3 (0-130)	24.2 (7.0-90.0)	0.01
Menopause status			
Post Pre Missing	409 (56.3%) 318 (43.7%) 1	105 (51.2%) 100 (48.8%)	0.20
Nodes			
Negative Positive Missing	285 (43.9%) 364 (56.1%) 79	97 (52.2%) 89 (47.9%) 19	0.05
ER			
Negative Positive missing	148 (20.4%) 578 (79.6%) 2	63 (31.0%) 140 (69.0%) 2	0.001
PR			
Negative Positive Missing	276 (38.0%) 450 (62.0%) 2	94 (46.5%) 108 (53.5%) 3	0.03
Her2			
Negative Positive missing	493 (68.4%) 288 (31.6%) 7	122 (75.8%) 39 (24.2%) 44	0.06
Triple negative			
Negative positive Missing	635 (87.7%) 89 (12.3%) 4	156 (82.1%) 34 (17.9%) 15	0.04
Chemotherapy			
No Yes missing	452 (63.3%) 262 (36.7%) 14	101 (49.5%) 103 (50.5%) 1	0.0004
Antracyclines in Chemotherapy			
No Yes	186 (71.0%) 76 (29.0%)	65 (63.1%) 38 (36.9%)	0.14
Tamoxifen therapy			
No Yes missing	377 (52.8%) 337 (47.2%) 14	104 (51.5%) 98 (48.5%) 3	0.74
Radio therapy			
No Yes Missing	245 (34.4%) 468 (65.6%) 15	64 (31.4%) 140 (68.6%) 1	0.43
Surgery			
Lumpectomy Mastectomy	555 (76.2%) 173 (23.8%)	170 (82.9%) 35 (17.1%)	0.04
Stage			
I II III Missing	153 (22.4%) 449 (65.7%) 81 (11.9%) 45	54 (27.6%) 124 (63.3%) 18 (9.2%) 9	0.24
Grade			
I II III Missing	69 (17.8%) 176 (45.5%) 142 (36.7%) 341	22 (15.1%) 58 (39.7%) 66 (45.2%) 59	0.20
Histology			
Ductal Lobular Other Missing	564 (77.7%) 52 (7.2%) 110 (15.2%) 2	172 (83.9%) 11 (5.4%) 22 (10.7%) 0	0.15

## Results

### Clinicopathological Characteristics

Individuals with a tumour with a medium/low RECQL protein levels had a higher prevalence of lymph node positivity (56.1% vs. 47.9%, p-value= 0.05), a larger mean tumour size (27.3 mm vs 24.2 mm, p-value = 0.01), a higher proportion of ER-positive tumours (79.6% vs. 69.0%, p-value = 0.001) and a smaller proportion of triple-negative tumours (12.3% vs. 17.9%, p-value = 0.04) compared to those with a high level of RECQL protein. There were no significant differences in HER2 status or tumour grade.

### Association of RECQL Protein Expression With Survival Among ER-Positive Patients

**Figure 1:**
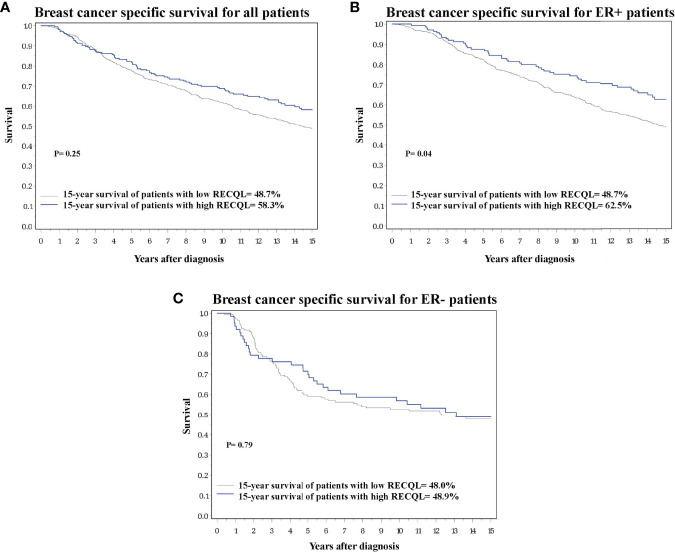
Breast cancer specific survival based on RECQL protein expression. **(A)** Breast cancer specific survival in the entire dataset based on RECQL protein expression. **(B)** Breast cancer specific survival in ER-positive patients based on RECQL protein expression. **(C)** Breast cancer specific survival in ER- patients based on RECQL protein expression.

Of the 933 tumour samples in the study, 78% (n = 728) had a medium/low RECQL protein level, and 22% (n = 205) had a high level. Patients with a high RECQL levels had superior 15-year survival compared to patients with medium/low RECQL levels (58.3% vs. 48.7%, HR = 0.79, 95%CI = 0.62-1.00, p-value = 0.05) (**Figure 1A**; **Table 2**). After adjustment for age at diagnosis, lymph node status, tumor size, chemotherapy, surgery, ER, PR and Triple Negative, a similar association was observed (58.3% vs. 48.7%, HR= 0.86, 95%CI= 0.67-1.11) but it was not significant (p-value= 0.25) (**Table 2**).

**Table 2:** Comparison of 15-year survival between patients with high versus medium/low RECQL levels.

Cohorts	Cases/total	Univariate *HR (95%CI) P	Multivariate HR (95%CI) P
All subjects			
Medium/Low RECQL High RECQL	356/728 79/205	1 0.79 (0.62-1.00) 0.05	1 0.86 (0.67-1.11) 0.25**^Δ^**
Subjects with ER-positive			
Medium/Low RECQL High RECQL	282/578 47/140	1 0.66 (0.48-0.90) 0.008	1 0.72 (0.52-0.98) 0.04**^Φ^**
Subjects with ER-			
Medium/Low RECQL High RECQL	74/148 31/63	1 0.96 (0.63-1.46) 0.85	1 1.07 (0.67-1.69) 0.79**^Φ^**
Subjects with ER-positive and Tamoxifen			
Medium/Low RECQL High RECQL	141/307 25/86	1 0.60 (0.39-0.92) 0.02	1 0.64 (0.41-0.99) 0.04**^Φ^**
Subjects with ER-positive and no Tamoxifen			
Medium/Low RECQL High RECQL	131/259 20/51	1 0.80 (0.50-1.28) 0.35	1 0.85 (0.52-1.38) 0.51**^Φ^**
ER- with Antracyclin-type chemo			
Medium/Low RECQL High RECQL	16/25 9/19	1 0.68 (0.30-1.54) 0.35	1 0.87 (0.33-2.24) 0.77**^Ω^**
ER- with no Antracyclin-type chemo			
Medium/Low RECQL High RECQL	58/123 22/44	1 1.03 (0.63-1.68) 0.92	1 1.20 (0.72-2.01) 0.48**^Ω^**
ER- with other type chemo			
Medium/Low RECQL High RECQL	25/55 13/28	1 0.92 (0.47-1.82) 0.81	1 1.13 (0.55-2.30) 0.75**^Ω^**
Post menopause			
Medium/Low RECQL High RECQL	226/409 48/105	1 0.79 (0.58-1.07) 0.13	1 0.80 (0.57-1.11) 0.18**^Δ^**
Pre menopause			
Medium/Low RECQL High RECQL	130/318 31/100	1 0.72 (0.49-1.07) 0.10	1 0.80 (0.53-1.20) 0.28**^Δ^**

*Adjusted for age at diagnosis.

^Δ^Adjusted for age at diagnosis, node, tumor size, chemotherapy, surgery, ER, PR and Triple Negative.

^Φ^Adjusted for age at diagnosis, node, tumor size, chemotherapy, surgery and PR.

^Ω^Adjusted for age at diagnosis, node, tumor size, surgery and PR.7

Multivariate survival analysis was conducted separately for ER-positive and ER-negative patients. Among ER-positive patients a higher 15-year survival rate was observed for those with a high level of RECQL protein (62.5% vs. 48.7%, HR = 0.72, 95%CI = 0.52-0.98, p-value = 0.04) (**Figure 1B**; **Table 2**). Among the ER-negative patients no difference was seen (48.9% vs. 48.0%, HR = 1.07, 95%CI = 0.67-1.69, p-value = 0.79) (**Figure 1C**; **Table 2**).

**Figure 2:**
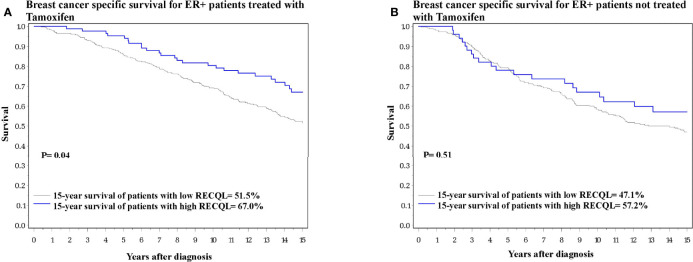
Breast cancer specific survival among ER-positive patients based on RECQL protein expression. **(A)** Breast cancer specific survival among ER-positive patients who received Tamoxifen based on RECQL protein expression. **(B)** Breast cancer specific survival among ER-positive patients who did not receive Tamoxifen based on RECQL protein expression.

We next investigated the effect of RECQL levels on ER-positive breast cancer patients subdivided by tamoxifen therapy. Among the ER-positive patients who received tamoxifen treatment, those who had higher RECQL protein levels had a better survival rate than patients with low RECQL levels (67.0% vs. 51.5%, HR = 0.64, 95%C I = 0.41-0.99, p = 0.04) (**Figure 2A**; **Table 2**). Among ER-positive patients who did not receive tamoxifen therapy, there was a smaller, non-significant, association between RECQL protein levels and survival (57.2% vs. 47.1%, HR = 0.85, 95%CI = 0.52-1.38; p = 0.5) (**Figure 2B**; **Table 2**).

**Figure 3:**
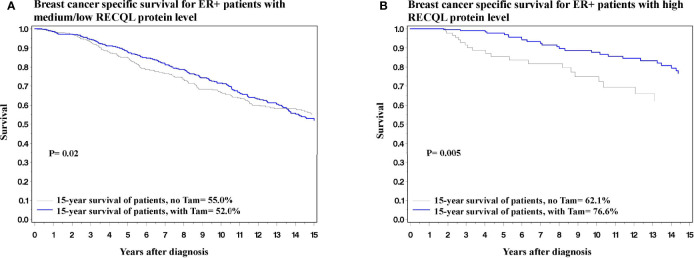
Breast cancer specific survival among ER-positive patients based on Tamoxifen treatment. **(A)** Breast cancer specific survival among ER-positive patients with medium/low level of RECQL protein based on Tamoxifen treatment. **(B)** Breast cancer specific survival among ER-positive patients with high level of RECQL protein based on Tamoxifen treatment.

In terms of 15-year survival, the benefit of tamoxifen treatment was greater in ER-positive patients with a high RECQL level (76.6% vs. 62.1%, HR = 0.37, 95%CI = 0.18-0.74, P = 0.005) than among ER-positive patients with a low/medium RECQ level (55.0% vs. 52.0%, HR = 0.73, 95%CI = 0.55-0.96, P = 0.02) (**Figure 3**; **Table 3**).

**Table 3:** Analysis of tamoxifen treatment influence on survival of ER-positive patients with medium/low and high RECQL protein levels.

Groups	Tamoxifen treatment	Case/total	Univariate HR(95%CI) P	Multivariate HR (95%CI) P
Medium/Low				
RECQL protein level	No Yes Missing	131/259 141/307 10/12	1 0.64 (0.50-0.82) 0.0005	1 0.73 (0.55-0.96) 0.02
High RECQL protein level	No Yes Missing	20/51 25/86 2/3	1 0.45 (0.24-0.82) 0.009	1 0.37 (0.18-0.74) 0.005

Univariate: adjusted by age at diagnosis only.

Multivariate: adjusted by age at diagnosis, node, tumor size, Chemotherapy (Yes or No), Surgery and PR.

## Discussion

We analyzed the association between RECQL protein levels and breast cancer survival among 933 breast cancer patients diagnosed from 1987 to 1999 in Toronto, Canada. Among unselected breast cancer patients medium/low levels of RECQL protein were associated with inferior disease-specific survival. Additional analysis revealed that this association was seen only among ER-positive patients, in particular among those who received endocrine therapy (Tamoxifen). Together, these data show that RECQL protein level is a prognostic factor for realizing a benefit from endocrine therapy.

Three other studies have investigated the association between RECQL protein or mRNA levels and breast cancer survival (1, 2, 34). The first study showed that in separate cohorts of 848 and 1977 breast cancer patients, lower protein and mRNA levels of RECQL were associated with worse prognosis; further analysis revealed that this association only held among the ER-positive patients (1). The second study also observed that among 774 breast cancer patients, lower mRNA levels of RECQL were associated with poor distant recurrence-free survival (HR: 2.77, p-value <0.001) and disease-specific survival (HR: 3.10, p-value <0.001). In a cohort of 322 breast cancer patients, low RECQL protein levels correlated with poor survival (HR: 2.12, p-value: 0.015); however, the authors did not compare this association among ER-positive versus ER- individuals (2). These studies also showed that lower RECQL protein levels were associated with poor clinicopathological characteristics (1, 2). We also observed that medium/low RECQL levels were associated with poor clinicopathological characteristics, such as a higher proportion of lymph node-positive tumours (56.1% vs. 47.9%, p-value= 0.05) and larger tumour sizes (27.3 vs 24.2, p-value= 0.01). However, other clinicopathological characteristics were better among the medium/low RECQL patients of our cohort; medium/low RECQL levels were associated with a larger proportion of ER-positive tumours (79.6% vs. 69.0%) and a smaller proportion of triple-negative tumour (12.3% vs. 17.9%), while there was no difference between the medium/low versus high RECQL patients in HER2 status, tumour stage, tumour grade, and tumour histology. Lastly, the third study was only focused on RECQL mRNA levels (34). They observed that a higher expression of RECQL mRNA was correlated with shorter relapse-free survival (RFS) (HR: 1.28, p-value <0.001, n= 3955) and post-progression survival (PPS) (HR: 1.32, p-value: 0.027, n= 414) in all breast cancers. However, higher expression of RECQL mRNA did not affect overall survival (OS) (HR: 1.04, p-value: 0.74, n= 1402) or distant metastasis-free survival (DMFS) (HR: 1.06, p-value: 0.57, n= 1747). The current body of evidence suggests that higher RECQL protein levels are associated with higher survival rates among breast cancer patients with ER-positive tumours.

The cellular mechanism of RECQL effect on the prognosis of ER-positive tumours might be explained by a recent study that explored the role RECQL plays in regulating ERα expression. According to this newly published study, in a helicase dependant manner, RECQL in cooperation with FOXA1 increases the chromatin accessibility at the regulatory site of the ESR1 gene (the gene encoding ERα), and a group of other ERα target genes (28). Therefore, higher levels of RECQL protein would increase ERα expression and its downstream effect. This is an important observation in breast cancer biology for two main reasons. First, higher ERα levels are associated with a better prognosis because ERα inhibits tumour invasiveness (35–38). Second, higher levels of ERα would enable a more desirable response to endocrine therapy (38–41). Therefore, higher RECQL level directly increases ERα level, and in turn could reduce tumour invasiveness and improve the response to endocrine therapy. Unfortunately, we did not have ER expression values in our cohort to compare with the RECQL data to confirm their positive correlation. The relationship between RECQL levels and ER expression, tumour invasiveness as well as endocrine treatment efficiency could be the subject of further research. More importantly, future studies should investigate how to induce RECQL expression in ER-positive patients to improve their prognosis, especially in patients with lower RECQL protein levels. The findings of such studies could have crucial clinical implications, especially considering that more than 70% of breast cancer cases are ER-positive (35, 42).

One question that remains to be answered is why patients with lower RECQL levels had lower survival, while lower RECQL levels result in lower levels of ERα, and as a result the mitogenic effects of estrogen and ERα should be reduced. Three factors should be considered in answering this question. First, lower ERα levels would result in higher invasiveness, reducing the survival rate (35, 36). Second, we observed a larger tumour size in patients with lower levels of RECQL protein, so either lower RECQL levels do not cause enough reduction in ERα levels to dampen the mitogenic effects of ERα, or there are other factors involved that not only compensated for the reduced mitogenic effects of ERα due to its reduction, but caused increased mitogenic effects and a larger tumour size. Third, it is likely that RECQL also impacts breast cancer patient survival through non-ERα dependent effects and through its role in maintaining the chromosomal stability and DNA damage response. Additional studies should be conducted to investigate if such factors exist, and if they do, what the mechanism by which they act as mitogens is, and what implications do they have for breast cancer and possibly other neoplasms.

## Conclusion

We have shown that higher RECQL protein levels are associated with improved ER-positive breast cancer-specific survival and better response to endocrine therapy with tamoxifen. Therefore, RECQL could be a prognostic and predictive candidate biomarker in ER-positive breast cancer patients responding to tamoxifen’s endocrine treatment.

## Data Availability Statement

The original contributions presented in the study are included in the article/**Supplementary Material**. Further inquiries can be directed to the corresponding author.

## Ethics Statement

This study has been approved by the ethics board committee at Women’s College Hospital. The patients/participants provided their written informed consent to participate in this study.

## Author Contributions

AM wrote the manuscript draft, AS did the laboratory assays, PS and VG did the statistical analysis, CC, SN-M, SS, SM, SN, and MA worked together in conceptualizing the study idea, contributing laboratory and biospecimen resources required for the study and writing the manuscript, AAS reviewed the IHC slides and helped with the revision of the manuscript. All authors contributed to the article and approved the submitted version.

## Funding

The study was funded by Canadian Institute for Health Research, grant# 152939.

## Conflict of Interest

The authors declare that the research was conducted in the absence of any commercial or financial relationships that could be construed as a potential conflict of interest.

## Publisher’s Note

All claims expressed in this article are solely those of the authors and do not necessarily represent those of their affiliated organizations, or those of the publisher, the editors and the reviewers. Any product that may be evaluated in this article, or claim that may be made by its manufacturer, is not guaranteed or endorsed by the publisher.

## Abbreviations

ER, Estrogen receptor; HR, Hazard ratio; IHC, Immunohistochemistry; TMA, Tissue microarrays.
