# Supplementary material for: High Expression of RECQL Protein in ER-Positive Breast Tumours Is Associated With a Better Survival
Source: Front Oncol. 2022 May 31;12:877617. doi: 10.3389/fonc.2022.877617 (PMC9195420; doi:10.3389/fonc.2022.877617)
Supplement: Supplementary file 1 [file DataSheet_1.pdf]

Supplementary Table 1: Distribution of median H-index across the cohort of 933 breast tissue samples

Median H-index Category	Number of breast tissue samples
0-30	5
31-60	34
61-90	73
91-120	223
121-150	135
151-180	118
181-210	141
211-240	75
241-270	39
271-300	90

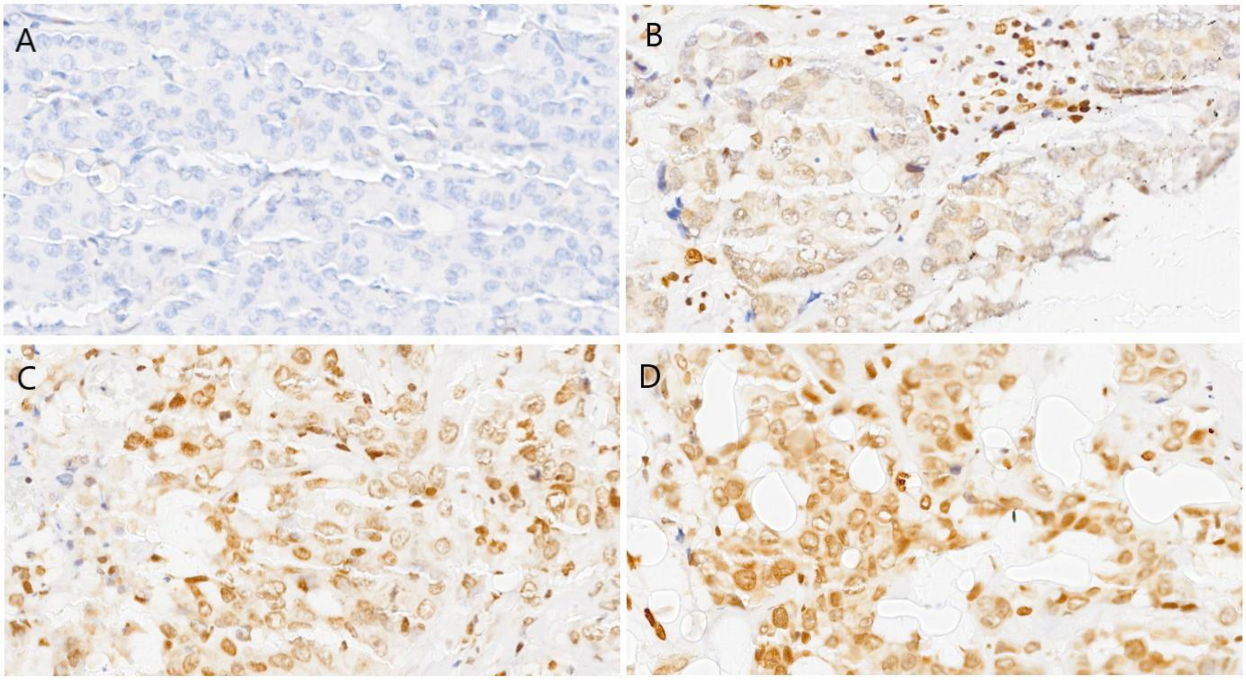

Supplementary Figure 1: Different intensities of the nuclear staining for RECQL protein (A: no staining (0), B: weak staining (1), C: moderate staining (2), and D: strong staining (3))
